# Acritarch-like Microorganisms from the 1.9 Ga Gunflint Chert, Canada

**DOI:** 10.1089/ast.2021.0081

**Published:** 2022-05-10

**Authors:** A.L. González-Flores, J. Jin, G.R. Osinski, C.J. Tsujita

**Affiliations:** ^1^ Department of Earth Sciences, University of Western Ontario, London, Canada.; ^2^ Institute for Earth and Space Exploration, University of Western Ontario, London, Canada.

**Keywords:** Cyst-like structures, Gunflint Chert microbiota, Complex unicellular bodies

## Abstract

Fossil evidence of eukaryotic life older than 1.8 Ga has long been debated because known fossils of that age usually lack cellular micro- and ultra-structures that bear strong affinities to eukaryotes. These include fossils of the ∼1.9 Ga Gunflint Chert microbiota that, despite being exceptionally well preserved, have suffered from cellular degradation, which poses challenges to studying their delicate cellular structures. In this study, we use an extended-focal-depth imaging technique, in combination with scanning electron microscopy, to document multiple types of large (10–35 μm diameter), cyst-like bodies based on distinctive details such as (1) radially arranged internal strands similar to those in some acritarchs and dinoflagellates; (2) regularly spaced long tubular processes, stubby pustules, and/or robust podia on the cell surface; (3) reticulate cell-wall sculpturing such as scale-like tubercles, pits, and ridges; and (4) internal bodies that may represent membrane-bound organelles. These micro- and ultra-structures provide strong morphological evidence for the presence of protists in the late Paleoproterozoic.

## 1. Introduction

Microfossils of suspected eukaryotes, both unicellular and multicellular, have been reported from Paleoproterozoic rocks from various locations around the world. Putative eukaryotic biomarkers have been reported from Neoarchean drill-core rocks as old as 2.7 Ga, but subsequent study has provided strong evidence for their origin to be a consequence of contamination from drilling (Brocks *et al.,* 1999; Rasmussen *et al.,* 2008; French *et al.,* 2015; Dacks *et al.,* 2016). Some tubular microfossils from rocks of ∼2.8–2.7 Ga of South Africa have been attributed, also questionably, to eukaryotes (Kaźmierczak *et al.,* 2016). Somewhat younger, possible eukaryotic fossils include the macroscopic, whip- to spiral-shaped *Grypania* from the Negaunee Formation of Michigan (originally thought to be ∼2.1 Ga by Han and Runnegar [1992] but later dated at 1874 ± 09 Ma by Schneider *et al.* [2002]) and the more morphologically variable fibro-radial bodies described from the ∼2.1 Ga Francevillian Group in Gabon, Africa (El Albani *et al.,* 2010, 2014, 2019). Similarly, a eukaryotic affinity has been a matter of debate for the microscopic, cyst-like bodies reported from the Paleoproterozoic, such as those of the Gunflint Chert, Canada (Barghoorn and Tyler, 1965; Cloud 1965; Cloud and Licari, 1968; Licari and Cloud, 1968; Barghoorn, 1971; Edhorn, 1973; Darby, 1974; Kaźmierczak, 1976, 1979; Krumbein, 2010). Although broadly similar to known eukaryotic cells morphologically, such microfossils have, thus far, failed to reveal well-preserved ultra-cellular structures (especially cross-linked features) one would expect for organic remains of protists (Cavalier-Smith, 2002).

Five different microfossils from the Gunflint Chert, which were originally identified as eukaryotes by Edhorn (1973), Darby (1974), and Kaźmierczak (1976, 1979), have since been reconsidered for the following reasons:

(a) The material described by Edhorn (1973) exhibited poor cellular preservation.
(b) The presence of a dark spot in the middle of the cellular structure, formerly suspected as evidence of a nucleus or organelles by Edhorn (1973), can be explained as a product of cellular degradation and cytoplasmic coagulation (Golubic and Hofmann, 1976).
(c) The bud-like projections in *Huroniospora* (Darby, 1974), which resemble similar features in fungi, can be explained as products of irregular growth patterns in the cell walls of cyanobacteria (Padmaja, 1972; Hirsch, 1974; Krumbein, 2010).
(d) The resemblance of *Eosphaera* to *Eovolvox,* which was thought to suggest affinities between these forms (Kaźmierczak, 1976), is largely superficial, as *Eosphaera* lacks a well-defined thick inner wall or any evidence of internal daughter colonies (Tappan, 1976).

Importantly, while geochemical data derived from *in situ* measurements of stable carbon isotopes in pyrobitumen and kerogen do not provide evidence for the presence of eukaryotic biomarkers in rocks 2.7 Ga or older (Rasmussen *et al.,* 2008), biomarkers corroborate the hypothesis that oxygenic cyanobacteria appeared by ∼2.15 Ga, followed by the appearance of eukaryotes by ∼1.78–1.68 Ga (Summons *et al.,* 1999). Despite the controversies surrounding the reliability of biomarkers and macrofossils as evidence for eukaryotic life, cyst-like microfossils have been considered reliable evidence for eukaryotes in rocks between 1.8 and 1.3 Ga, especially those with a well-preserved cytoskeletal cell wall and complex ornamentation (Meng *et al.,* 2005; Knoll *et al.,* 2006). Indeed, cyst-like microfossils described from the nonmarine deposits of the ∼1.2 Ga Torridonian Supergroup of Scotland are currently considered the oldest known protists (Strother *et al.,* 2011). In most cases, however, the identification of these ancient fossils as protists remains a formidable challenge, mainly due to cell body degradation, which renders it difficult, if not impossible, to recognize potential cell nuclei and organelles (if they were indeed present at all). As a result, studies on these forms have typically been made via comparisons of their overall morphology with cyst-like fossils of Phanerozoic age, such as acritarchs, dinoflagellates, or other unicellular algae. With regard to fossil eukaryotes, the diverse and well-preserved acritarch assemblages of Ediacaran rocks of Australia (Willman and Moczydłowska, 2008) provide a good basis for comparison and identification of older acritarchs and cyst-like forms. Such protist cysts are characterized by large cells (30 μm or larger in diameter) with multilayered walls (often folded and/or wrinkled in preservation) and an outer surface ornamented with a reticulate, polygonal, or other sculptured pattern and prominent hollow processes.

**FIG. 1.**
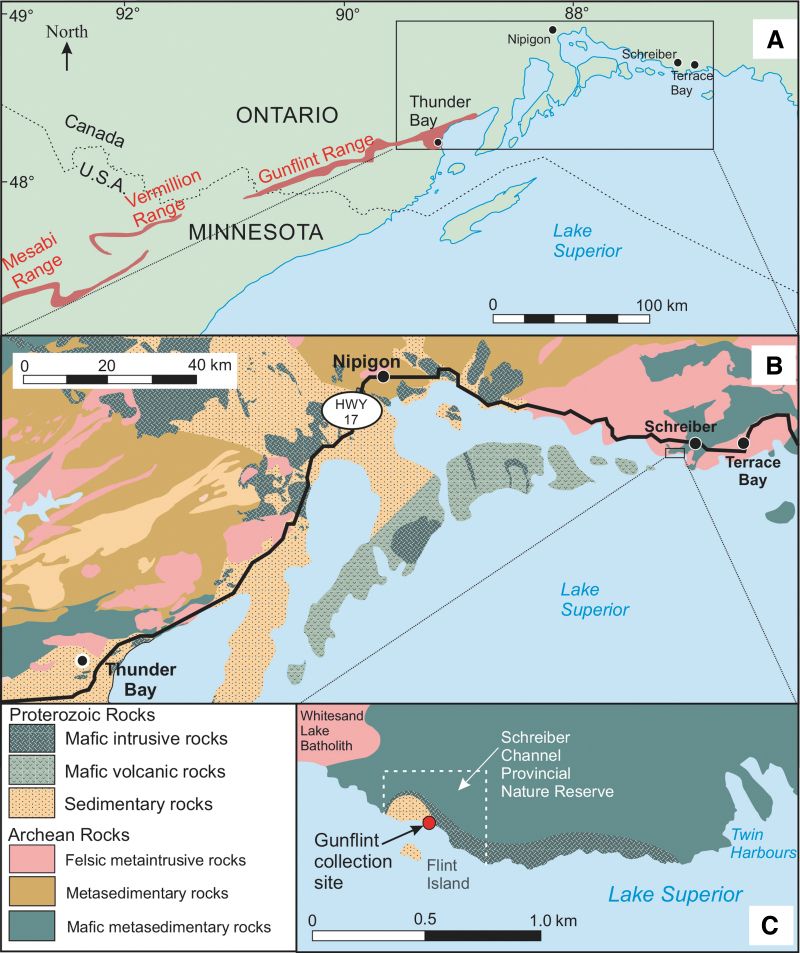
Location map of the Gunflint Chert. (**A**) Map of the northern Lake Superior region showing the Proterozoic Mesabi, Vermillion, and Gunflint iron ranges. (**B**) Enlarged view of area indicated in (A) at the northern tip of the Gunflint Iron Range, showing the distribution of Proterozoic and Archean rocks. The Gunflint Formation is included in a succession of Proterozoic sedimentary rocks underlain by Archean metamorphic rocks. (**C**) Enlarged view of area indicated in (B) showing the location of the collection site for Gunflint Chert samples used in this study. The collection site is a coastal outcrop of a small outlier of the Gunflint Chert located within Schreiber Channel Provincial Reserve, approximately 10 km southwest of Schreiber, Ontario (GSC Locality No. 78417, 87°20'48"W, 48°47'50"N). The samples were collected by Hans Hoffmann in 1967 prior to the establishment of the reserve (1979). (A) Modified from Cannon *et al.* (2008); (B) and (C) modified from Magnus (2015).

Well known for its diverse microbiota, the richly fossiliferous and usually black to dark-gray rocks of the Gunflint Chert occur in the Gunflint Iron Range along the northern shore of Lake Superior, Canada, from the northern tip of Lake Gunflint (Cook County), Minnesota, to the district of Thunder Bay, Ontario (Fig. 1A). Rocks of the Gunflint Iron Range comprise Proterozoic metasediments that overlie Archean metasedimentary, metavolcanic and granitic intrusive rocks (Fig. 1B). Metasedimentary strata of the Paleoproterozoic Animikie Group are divided into three formations. These are, in ascending order, The Kakabeka Conglomerate, the Gunflint Formation, and the Rove Formation. The Gunflint Formation conformably overlies the Kakabeka Conglomerate (Fralick and Barrett, 1995) and, in turn, is overlain conformably by the metamudstones of the Rove Formation (Morey, 1967, 1969). The lower part of the Gunflint Formation contains the microfossil-rich *Gunflint Chert* unit, which is the focus of this study. In the discussion below, the term “Gunflint Chert” refers to this specific unit. Nd/Sm dating yielded an age range for this unit to be 2.08–2.11 Ga based on whole rock and 1.86–1.99 Ga for ashes (Stille and Clauer, 1986; Gerlach *et al.,* 1988; Fralick *et al.,* 2002). The approximate upper age limit of the Gunflint Formation is based on the U-Pb age of 1.87 Ga (Morey, 1969; Fralick *et al.,* 2002) for the upper Gunflint Formation and the overlying Rove Formation. The Gunflint Formation is a 120 m thick succession of metasedimentary strata that includes siliciclastic, carbonate, chert, and iron formation (taconite) units, with stromatolitic horizons (Fralick *et al.,* 2002). The Gunflint Formation is interpreted to have been deposited on the inner, shallow-water part of a marine platform influenced by strong wave and tidal activity (Wacey *et al.,* 2012).

The exceptional quality of microfossil preservation in the cherty beds of the Gunflint Chert make this unit an important source of Proterozoic fossil data (Wacey *et al.,* 2012). Such preservation is attributed to multiple factors, which include the following: (1) a very low grade of metamorphism (with burial temperatures of ∼150°C); (2) the protective attributes of the preservation media (chert and microquartz) against post-depositional damage (Winter and Knauth, 1992; Marin *et al.,* 2010); and (3) hydrothermal circulation of oxygenated fluids that may have caused an increase in temperatures without having induced any recrystallization of the microquartz matrix (Alleon *et al.,* 2016). The best-preserved microfossils yet found in this unit occur in a small outlier of the Gunflint Chert located on the Lake Superior shoreline southwest of Schreiber, Ontario (Fig. 1B, 1C), where the effects of metamorphism were minimal (Barghoorn and Tyler, 1965).

Microfossils of the Gunflint biota were initially proposed to be the remains of eukaryotes, with affinities that range from unicellular algae to radiolarians (Edhorn, 1973). These claims, however, were later refuted, and the microfossils in question were reinterpreted as prokaryotes (Awramik and Barghoorn, 1977). Although cherts of the Gunflint Chert have not yielded undisputed acritarchs, relatively large organic-walled spherical microfossils, such as *Eosphaera,* have indeed been reported (Barghoorn and Tyler, 1965; Edhorn, 1973). Awramik and Barghoorn (1977) assigned the Gunflint microbiota to 16 taxa that were variously categorized as cyanophytes, budding bacteria, and organisms of unknown affinities. It is the latter group that is the main focus of this study.

## 2. Material and Methods

The samples of Gunflint Chert used in this study were loaned from the Geological Survey of Canada (GSC) and originally collected by the late Hans Hofmann in 1967 (see Hofmann, 1971) from a coastal outcrop (GSC Locality No. 78417, 87°20'48"W, 48°47'50"N) approximately 10 km southwest of Schreiber Beach, Ontario, Canada (Fig. 1). This exposure is located in a very small outlier of the Gunflint Chert (Fig. 1C). Among the samples collected from this locality, only sample GSC24380 was used for this study, and a total of six thin sections, numbered GSC24380 (2), GSC24380d (2), and GSC24380e (2), were prepared from the sample.

Optical microscopy was performed with a Zeiss Axioscope with a 100 × oil lens and a 1.6 × intermediate lens, achieving a combined 160 × optical magnification. Confocal image sequences were acquired manually and processed by using the extended depth of focus (EDF) function of the Nikon NIS Elements imaging software package (ver. 4.1 and ver. 5.02). Thus, a Z-series of confocal images, with approximately equal steps in depth, was combined into a single EDF image (*e.g.,* see the Supplementary Material). For spherical objects, EDF images of the lower and the upper hemispheres were created separately to avoid overlap of cell surface features of the two hemispheres. Once the images were obtained through the EDF process, the shade and contrast of the images were enhanced with Adobe Photoshop C19. Based on the images, a qualitative analysis of these was done to identify and differentiate the different types of organisms discussed in the later section.

The measurement of cell diameters of the different bodies was acquired through Nikon NIS Elements imaging software and Adobe Photoshop C19. A total of 900 EDF images were acquired, with 300 each from three of the most fossiliferous thin sections (GSC24380c, GSC24380d, and GSC24380e). Each of such images were derived from a Z-series of 5–17 stacked photos.

**FIG. 2.**
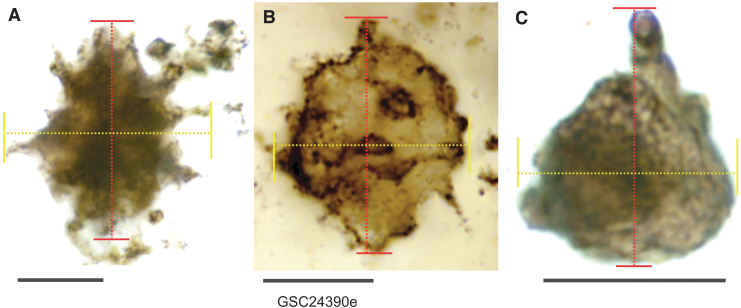
Diameter measurements of CUBs. For every CUB type, two diameters were measured: longitudinal diameter (D1, red) refers to the maximum diameter, and transversal diameter (D2, yellow) at perpendicular angle to D1. (**A**) Measurements of CUB Type 1. (**B**) Measurements of CUB Type 2. (**C**) Measurements of CUB Type 3, all from thin section GSC24380e. Scale bar = 10 μm.

In this study, image acquisitions are focused on the relatively large, complex cell bodies, although filamentous and coccoid bacteria were also imaged on each sample for the purpose of size and distribution pattern comparisons among the various microfossil forms present. Two parameters were defined when measuring the diameter (Fig. 2): longitudinal diameter (D1, typically the maximum diameter, including the process and podia) and transversal diameter (D2, usually shorter and measured at perpendicular angle to D1).

Scanning electron microscopy (SEM) imaging for sample GSC24380d was performed with the LEO (Zeiss) 1540XB FIB/SEM at the Nanofabrication Facility (University of Western Ontario) at 1.00kV. The sample of chert was lightly etched in HF (5%) then sonicated in deionized water for 15 min and then allowed to dry on a hotplate set at 115°C. The chert was then placed in a beaker of heptane, covered with a watch glass, and allowed to soak for 60 min. The sample was then removed and dipped in a second beaker of fresh heptane, which served as the final rinse. The chert was then allowed to air-dry overnight in a fume hood. The purpose of the heptane was to de-grease the sample before SEM imaging.

The possibility of the observed spherical cellular bodies having been emplaced on the thin sections via sample contamination is precluded by three lines of evidence:

(1) Through confocal examination and imaging of the thin sections, the cyst-like bodies documented in this study are unequivocally embedded within the chert (30–50 μm thick), not on either bottom or top surfaces of the thin sections.
(2) Some of the spherical bodies co-occur in loose clumps with other Gunflint microorganisms, such as the ubiquitous cyanobacterium *Gunflintia* and small spheres of *Eosphaera.*
(3) SEM imaging shows that some of the spherical bodies are filled with microcrystalline quartz that also forms parts of the otherwise cherty (cryptocrystalline) matrix of the rock. This observation of primary preservation of the microorganisms corroborates the TEM study of the Gunflint Chert by Moreau and Sharp (2004), who demonstrated an organic influence on microquartz precipitation at microfossil-matrix boundaries.

## 3. Results

At least 16 morphospecies of microorganisms have been previously identified from the Gunflint Chert (Awramik and Barghoorn, 1977). For this study, however, we focus on the larger “complex unicellular bodies” (CUBs) with complex cellular structures, as opposed to their co-occurring, smaller-celled filamentous and coccoid bacteria. As confirmed in this study, the filamentous forms are the most abundant fossils in the Gunflint Chert, with individual cells having diameters of 0.49–0.58 μm and forming filaments up to several hundred micrometers in length (*cf.* Barghoorn, and Tyler, 1965). The next most abundant microfossils are coccoid bacteria, characterized by spheroidal to ellipsoidal shape, diameters of 1.0–1.7 μm, variable cell wall thickness, and a pitted or granular surface without protruding processes (see Awramik, 1976). The coccoid forms differ from the CUBs in being much smaller and lacking surface processes or intracellular structures. Based on images from a microfossil-rich chert sample of the Gunflint Chert (GSC24380), we recognize three types of CUBs and one type of “multicellular” body, as described below.

### 3.1. CUB Type 1: Spherical cysts with numerous fine processes

**FIG. 3.**
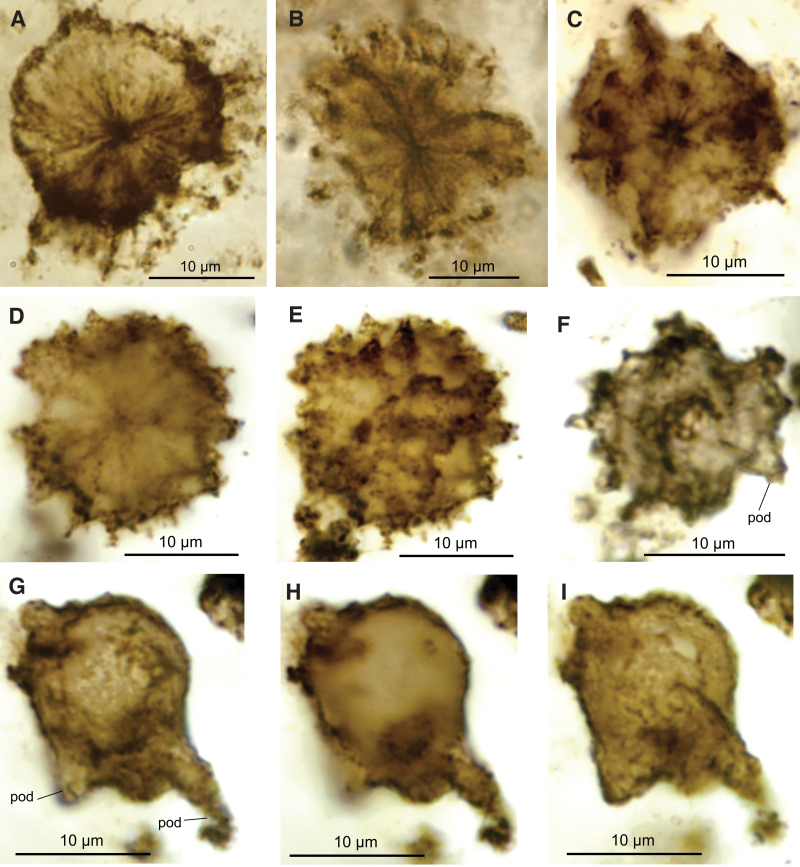
Photomicrographs of CUBs. (**A**, **B**) CUB Type 1, cells with a spheroidal body bearing radial strands, inter-strand membrane, and delicate processes projecting from cell surface, GSC24380d, thin section no. 2. (**C**–**E**) CUB Type 2, two spheroidal cells with numerous hornlike pustules (or podia) projecting from surface, GSC24380e, thin section no. 2. (**D**) and (**E**) are extended depth of focus (EDF) images through the “equatorial-center” zone (D) and one hemisphere (E) of the same CUB (see the Supplementary Material for more detailed explanation). (**F**–**I**) CUB Type 3, two ovoidal cells with robust podia (labeled “pod”) of uneven sizes; (**F**) GSC24380d, thin section no. 2; (**G**–**I**) GSC24380e, thin section no. 2, three EDF views of the same cell in two hemispheres (G, I) and “equatorial-center zone (**H**).

These are the best-preserved and most common CUBs examined in this study, with a high level of their original complexity preserved. Their overall shape resembles a star or spoked wheel. Visible between the “spokes” are what appear to be remnants of a thin retractable membrane (Fig. 3A, 3B). Most of the organisms have a central structure, from which long endofilaments radiate outward, forming free hollow-branched processes beyond the membrane (Fig. 3A). The weblike membrane between the radiating endofilaments is presumed to be the shrunken remnants of a cell wall. *CUB Type 1* cells range in size from 14 to 25 μm but most commonly from 18 to 22 μm (see Fig. 5). *CUB Type 1* differs from Type 2 and Type 3 in cell structure, especially in that its membrane is supported by thin, radiating, and hollow filaments.

### 3.2. CUB Type 2: Spherical cells with horny pustules

**FIG. 4.**
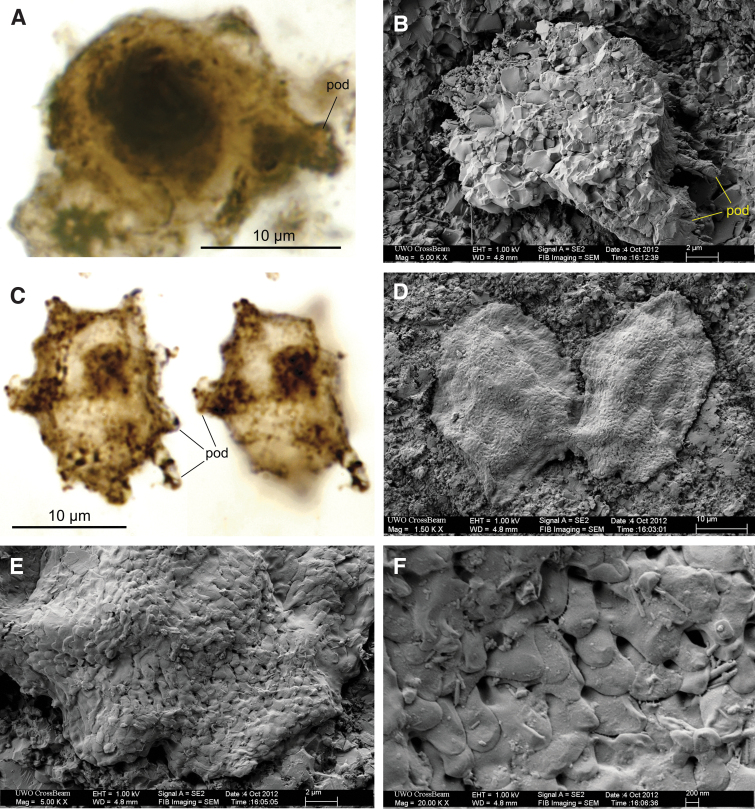
Extended depth of focus (EDF) optical and SEM images of CUB Type 3. (**A**) EDF view of cell with a large central dark body and a robust podium (“pod”), GSC24380d, thin section no. 2. (**B**) SEM image of internal mold (consisting of microquartz) after light HF etching, showing a large podium and smaller ones (labelled “pod”; note large podium similar to that in Figs. 3G–3I and 4A), GSC24380d, small rock block. (**C**) Two EDF views (top and bottom sides) of same cell with scale-like ornaments and podia, GSC24380e, thin section 2. (**D**–**F**) SEM images of two partly squashed cells (**D**) similar in shape to (C), and details of their imbricated, scale-like surface ornaments (**E**, **F**).

**FIG. 5.**
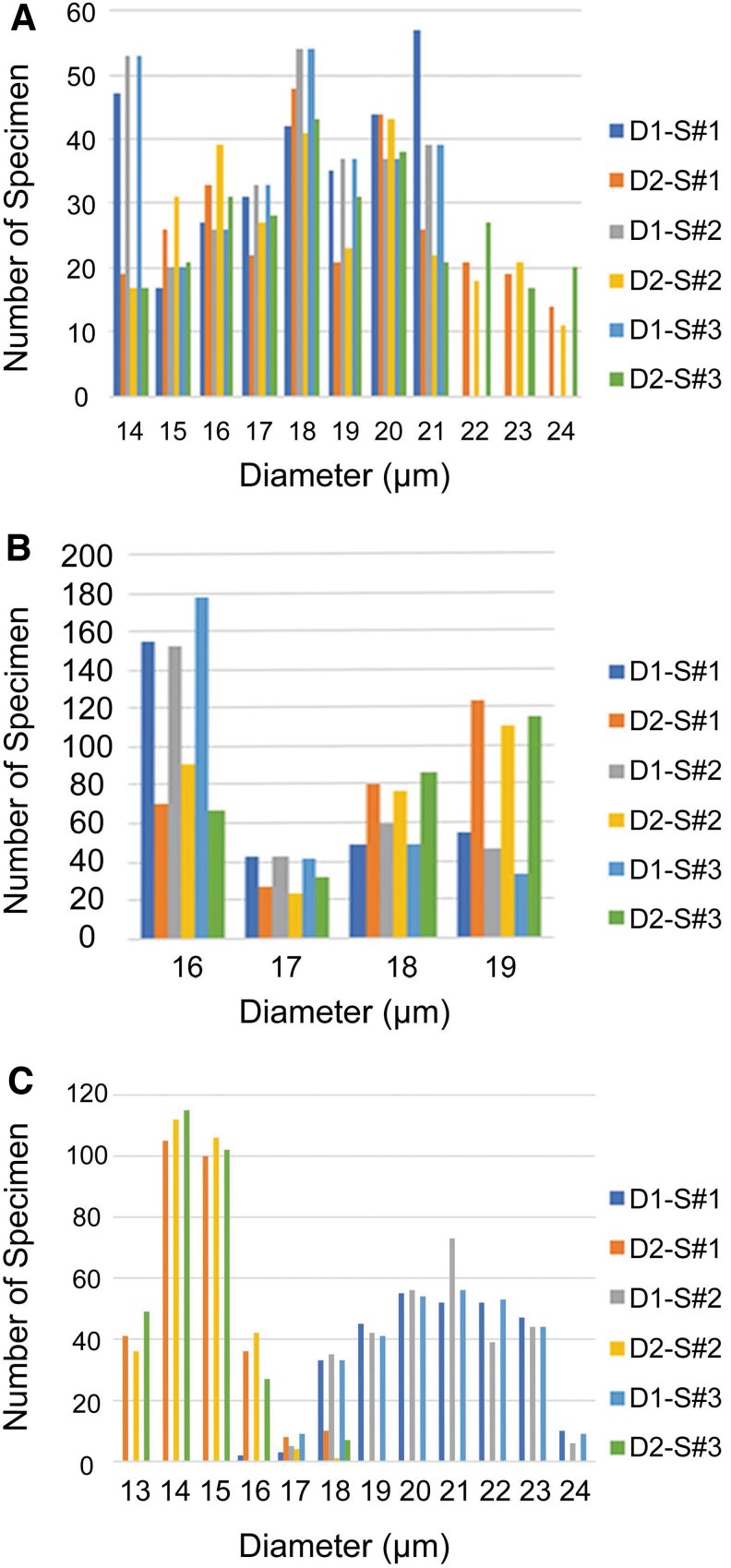
Histograms of the distribution of longitudinal and transversal diameters of CUB Types 1–3, based on 900 EDF images of cells acquired at × 160 magnifications from three thin sections (see the Materials and Methods section for details). (**A**) The diameter distribution of CUB Type 1, ranging from 14 to 24 μm. (**B**) Diameters for CUB Type 2, the most spherical-like features with values ranging from 16 to 19 μm. (**C**) Diameters for CUB Type 3, with values ranging from 13 to 24 μm, inclusive of podia.

These cells are relatively large spherical bodies that show rather numerous, stubby horns or pustules on the cell surface (Fig. 3C–3E; see also the Supplementary Material). The cysts have an average diameter of 16–19 μm (Fig. 4B). Despite its relatively large size among Gunflint microorganisms, this type does not seem to have been reported in previous studies. *CUB Type 2* cells are subspherical and show the smallest variation between D1 and D2 diameters as well as in overall specimen size. They have a transversal diameter range from 16 to 18 μm and a longitudinal diameter from 16 to 19 μm, with a higher concentration toward the lower end of the size range (around 16 μm for D1 and D2; see Fig. 5). In conventional optical microscopy, the cells can be seen to bear radiating “spokes” or strands that merge at the cell center and extend from the center to the cell wall. Type 2 CUBs are the second most common in the samples examined, with more than 1700 individuals observed in this study.

### 3.3. CUB Type 3: Irregular cells with robust podia

This cell type is sub-spheroidal to sub-ovoid in shape and bears a relatively small number of large, hollow, variously sized processes or pustules (Fig. 3F–3I), called “podia” here for simplicity. The longitudinal diameter ranges from 13 to 24 μm, averaging 15.27 μm (Fig. 5). Available data indicate that the podia are of variable sizes and irregularly distributed on the cell surface. One particularly well-preserved specimen (Fig. 4B) suggests that small processes are common on the cell surface in addition to the large process. The variable length and diameter of the podia, however, may have been due, at least partly, to preservational factors. Some well-preserved podia measure ∼8 μm in length (Figs. 3G–3I and 4A–4C). Some shorter podia are assumed to be remnants of longer structures, as their hollow structure is evident at their broken terminations (Figs. 3G–3I and 4C). Other smaller podia, cylindrical to conical in shape, hollow, range from 2 to 4 μm in length, up to 1 μm in diameter, and commonly display a rounded or slightly tapering end (*e.g.,* “pod” in Fig. 4C).

*CUB Type 3* cells are translucent like other types but usually darker, making it difficult to discern the internal features as well as those present in the cell membrane that are easier to observe in the other two types.

In overall morphology, some of the *CUB Type 3* cells resemble those of *Germinosphaera alveolata* (Miao *et al.,* 2019) from the late Paleoproterozoic Chuanlinggou Formation of China, which were reported recently also from the Mesoproterozoic Fort Confidence Formation, Dismal Lakes Group (1590–1270 Ma) in Arctic Canada (Loron *et al.,* 2021), as having a spheroidal cell body, with a large robust process that is hollow, broad-based, and slightly tapering toward the end. A more striking similarity is the imbricated, scale-like ornaments on the cell surface, present in both the Arctic Canada and Gunflint Chert specimens (compare Loron *et al.,* 2021, fig. 7.8–7.10, with Fig. 4D–4F of this study). The only notable difference is the cell size; the cells from the Mesoproterozoic of Arctic Canada were described by Loron *et al.* (2021) to have a range of 25.9–57.0 μm, although a small ovoidal specimen they illustrated has a short diameter of ∼20 μm and a long diameter of ∼24 μm (excluding the process).

### 3.4. “Multicellular” bodies

**FIG. 6.**
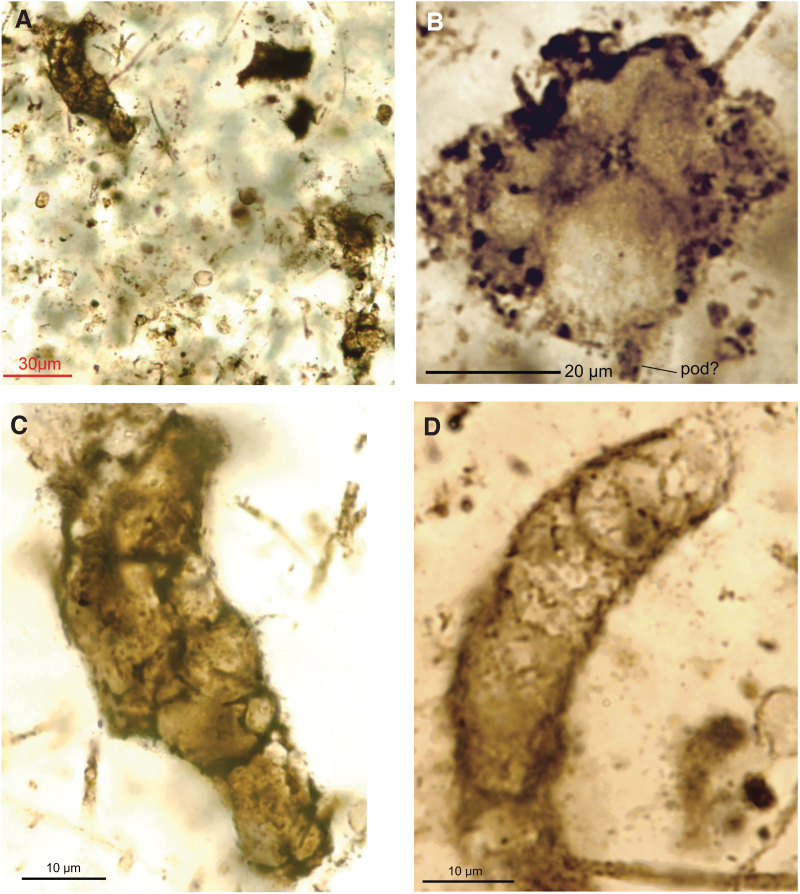
“Multicellular” bodies. (**A**, **C**) Brocken pieces of a rope-like organisms with well-integrated cells of various sizes (C enlarged from upper-left corner of A), with thin, threadlike, filamentous cyanobacteria in the background, GSC24380d, thin section no. 2. (**B**) Four-celled structure resembling fragment in lower-right corner of (A), GSC24380e, thin section no. 2. (**D**) Cylindrical form with uniserial, longitudinally stacked cells, GSC24380e, thin section no. 2.

This group of microfossils consists of various congregations (*i.e.,* clusters or clumps) of bodies. They appear somewhat darker in color compared to the other types and have a distinct reticulate pattern visible on the wall of some cells that helps differentiate individual bodies within a clump. These clumps may exceed 50 μm along the long axis (*e.g.,* Fig. 6A, 6C, 6D). Some of the specimens of this type show superposition of the cells at different levels of the *z*-axis as well as patterns with a lighter coloration within them, giving the appearance of an amorphous black mass. In some specimens, the cells show a higher degree of integration, resembling a multicellular organism with a uniserial (Fig. 6D) to multiserial (Fig. 6A–6C) organization of cells. It is difficult to discern the characteristic podia (as in CUB Type 3) because of the reduction of transparency by overlapping cells, but they may be present in some specimens (*e.g.,* “pod?” in Fig. 6B). Crudely tubular “multicellular” bodies are commonly 10–15 times thicker than their co-occurring, delicate, filamentous bacteria (*e.g.,* Fig. 6C, 6D).

## 4. Discussion

Each of the thin sections examined in this study contains a wide assortment of microorganisms, without any observable patterns of concentration for any given type of CUBs. Individual cells of CUBs examined in this study show variable shapes, with complex ornaments on the cell wall surface. Some characteristics of the CUBs show various degrees of resemblance to certain unicellular protist cell features.

### 4.1. Eukaryote-like characteristics of the Gunflint CUBs

In this study, two characteristics of the Gunflint CUBs are considered eukaryote-like: cell size and the presence of complex cell-wall features. Although most bacteria and archaea fall into a size range of 0.5–5.0 μm (Awramik and Barghoorn, 1977; Shimkets, 1998), some prokaryotes can attain larger sizes. Some Archean microfossils interpreted as cyanobacteria (*e.g.,* Altermann and Kazmierczak, 2003), or simple, biogenic microstructures (Sugitani *et al.,* 2010), for example, can reach 10–40 μm in diameter. Individual cells of CUBs examined in this study have diameters ranging from 13 to 25 μm. This size range is smaller than that of most of the younger acritarch or other eukaryote-like microfossils of Paleo- to Mesoproterozoic age (commonly >50 μm in diameter; *e.g.,* Agić *et al.,* 2017; Miao *et al.,* 2019; Loron *et al.,* 2021), but this smaller size range is well within that of modern unicellular green algae (Kaźmierczak, 1976).

As noted earlier, the Mesoproterozoic *Germinosphaera* from Arctic Canada with typical eukaryotic features (robust process and scale-like cell wall ornaments) usually have a size of 26–57 μm but can be as small as ∼20 μm in diameter (Loron *et al.,* 2021), which overlaps with the size of larger CUBs in this study. A wide range of variation in cell/cyst size within a single species or genus has been observed in much younger organic-walled microorganisms, such as the acritarch *Leiosphaeridia* from the Silurian (Wenlock) of Lithuania (Spiridonov *et al.,* 2017). These authors noted that, within a short geological time of ∼0.5 Myr, the average cyst diameter of *Leiosphaeridia* differed by about 5 times between cold and warm episodes, with a total range of cyst size from 10 to 150 μm. It is difficult to decipher the climate and ocean water temperature change for the 1.9 Ga Gunflint Chert, but the generally smaller cell size of Gunflint CUBs may have been, in large part, a reflection of their early evolutionary stage, if these microorganisms are indeed eukaryotes.

The Gunflint cyst-like cells appear to have had a strong and flexible wall, as indicated by the absence of fractures despite wrinkling and folding (Figs. 3 and 4). The reticulate-patterned surface ornamentation, which includes well-defined processes, perforations/pits, ridges, and stubby pustules, large podia, and imbricated scale-like ornaments, is a common characteristic of protist cells (*e.g.,* Awramik, and Barghoorn, 1977; Agić *et al.,* 2017) but is unknown in prokaryotes. These complex organic-walled bodies are regarded by some as possessing truly diagnostic characters of eukaryotes, as the formation of these features requires an endomembrane and cytoskeleton, which are known only in eukaryotes (Cavalier-Smith, 2002). Such complex cell-wall structures have been key to the interpretation of Paleo- to Mesoproterozoic eukaryotic microfossils in many recent studies (Agić *et al.,* 2017; Miao *et al.,* 2019; Loron *et al.,* 2021).

Many morphotypes of the complex Gunflint microfossils resemble acritarchs from the Ediacaran strata of the Officer Basin, Australia, studied by Willman and Moczydłowska (2008). These authors reported a total number of 23 different acritarch species from the Officer Basin. The main difference between these and the Gunflint CUBs is size. The Ediacaran organisms are usually larger than 50 μm in diameter, whereas the CUBs in the Gunflint chert do not exceed 35 μm. However, they share similar morphology, as both sets of organisms show a complex wall-surface ornamentation, filamentous structures, and dark spots at their center (Awramik and Barghoorn, 1977; Darby, 1974; Kaźmierczak, 1976, 1979).

Acritarchs are one of the earliest groups of eukaryotes recognized in the fossil record (Knoll, 2015; Moczydłowska *et al.,* 2011; Butterfield, 2015), and there is some evidence that the complex cells in the Gunflint Chert are eukaryotic. For example, Type 1 CUBs have a similar cell shape and surface ornaments to those of *Alicesphaeridium medusoideum* (Willman and Moczydłowska, 2008), although the latter from the Ediacaran of Australia are notably larger in size (>100 μm) and show better developed radial filaments than those of the star-shaped Gunflint microfossils.

*CUB Type 1* are the largest and internally complex unicellular bodies among the Gunflint Chert microfossils. The projections (Fig. 3A, 3B) are comparable to the microtubule arrangements in the cellular cytoskeleton of a protist (Menzel and Elsner-Menzel, 1990). The Gunflint biota also contains more delicate radiate structures that lack a well-defined central cell body and are commonly smaller than *CUB Type 1* described here. These small star-shaped features most likely represent cyanobacteria, such as *Trichodesmium* (Cloud, 1965; Rubin *et al.,* 2011).

Based on the images obtained in this study, *CUB Type 1* also resembles the Mesoproterozoic *Shuiyousphaeridium* from the in Beidajian Formation of North China (Meng *et al.,* 2005) and several Ediacaran acritarch genera from the Officer Basin of Australia (Willman and Moczydłowska, 2008), especially in their spherical cell body with numerous, long processes.

Some of the bodies resemble the “desmid-like” or “radiolaria-like” organisms noted by Edhorn (1973). Relatively delicate processes show an irregular texture, thicker proximally, and thinner distally, like those in the Ediacaran acritarch *Tanarium* from Australia (Willman and Moczydłowska, 2008). In some cells, the processes appear to spread into the meshwork (Fig. 3A), like those in the Ediacaran acritarch *Appendisphaera* (Willman and Moczydłowska, 2008). In others, the processes appear more robust, with relatively few of these showing the random curvature observed in *Tanarium* from the same Ediacaran assemblage.

In their cell shape, size, and ornamentation, Type 2 CUBs are similar to *Gambierdiscus toxicus,* a modern dinoflagellate (Knoll, 2015). Some specimens show dark-brown bodies with rather clear boundaries inside the cell, which may be preserved nucleus or organelles. Reticulate sculpturing is visible on the wall of some cells like those observed in BSE images, with regularly spaced pustules, pits, and ridges.

Apart from their relatively small size, some cells of *CUB Type 3* with multiple podia on the cell surface (“pod” in Fig. 4C) resemble some much younger (Ediacaran) acanthomorph acritarchs (Willman and Moczydłowska, 2008). The prominent single large podium and scale-like cell surface ornaments in some other CUB Type 3 cells (Figs. 3G–3I and 4A, 4B) are characteristic of *Germinosphaera alveolata* of late Paleo- to Mesoproterozoic age (see discussion above). All these complex cell-wall structures have been regarded as eukaryotic characteristics (for recent summary, see Agić *et al.,* 2017; Miao *et al.,* 2019; Loron *et al.,* 2021).

## 5. Conclusions

Recent studies have interpreted well-preserved acritarchs and other complex organic-walled microfossils of Proterozoic age as eukaryotes, pushing back the record of these fossils ∼1.8 Ga (*e.g.,* Montenari and Leppig, 2003; Willman and Moczydłowska, 2008; Moczydłowska *et al.,* 2011; Butterfield, 2015; Agić *et al.,* 2017; Miao *et al.,* 2019; Loron *et al.,* 2021). Putative eukaryotic microfossils have been reported from the Archean (Kaźmierczak *et al.,* 2016), although the age is in general agreement with predictions of the genome/molecular timescale (Hedges *et al.,* 2001, 2004).

The present study on the Gunflint Chert using extended-focal-depth optical microscopy and SEM imaging of the complex unicellular bodies (CUBs) has demonstrated a number of cellular morphologies similar to those of younger Proterozoic microfossils reported to be of eukaryotic affinity.

(1) Relatively large cell size. The CUBs range from 10 to 35 μm in diameter (commonly 13–25 μm), which is considerably larger than the coccoid cyanobacteria in the same samples (usually ∼5 μm). They are, however, generally smaller than the Proterozoic acritarchs, though the size of larger Gunflint CUBs overlaps with the smaller forms of Mesoproterozoic *Germinosphaera* interpreted by various authors as eukaryote.
(2) Complex surface ornaments. The reticulate-patterned pits and tubercles and variously shaped processes are not present in the coccoid cyanobacteria in the Gunflint Chert. These cell surface ornaments bear a high degree of similarity to those in acritarchs and other organic-walled microfossils regarded as eukaryotes.
(3) Intracellular dark bodies. The radiating filaments and well-delimited dark bodies inside the CUB cells may have been the residue of nuclei or organelles.

## Supplementary Material

Supplemental data

## Abbreviations Used

CUBs: complex unicellular bodies
D1: longitudinal diameter
D2: transverse diameter
EDF: extended depth of focus
GSC: Geological Survey of Canada
SEM: scanning electron microscopy
