# Supplementary material for: Acritarch-like Microorganisms from the 1.9 Ga Gunflint Chert, Canada
Source: Astrobiology. 2022 May 10;22(5):568–78. doi: 10.1089/ast.2021.0081 (PMC9125578; doi:10.1089/ast.2021.0081)
Supplement: Supplemental data [file Suppl_Material.pdf]

Acritarch-like microorganisms from the 1.9 Ga Gunflint Chert, Canada

A. L. González-Flores[1,2], J. Jin[1], G. R. Osinski[1,2], and C. J. Tsujita[1]

[1]Department of Earth Sciences, Western University, London, ON N6A 3K7, Canada.
[2]Institute for Earth and Space Exploration, Western University, London, ON N6A 3K7, Canada.

Correspondence:

Ana Laura González Flores, Department of Earth Sciences, Biological and Geological Sciences Building, Western University, 1151 Richmond St, London, ON N6A, Canada
Phone: +52 (444) 245-9860
Email: agonzlez@uwo.ca

Supplementary Document

Extended focal depth optical imaging

In this study, images of the Gunflint Chert microfossils were acquired using a Zeiss Axioscope. Maximum magnification was achieved with a 100x oil lens and a 1.6x intermediate lens, obtaining a combined 160x optical magnification, which applies to nearly all the images presented in this paper. At this magnification, it is impossible to bring every part of a microfossil (with a diameter ranging from 13 to 25 µm in diameter) into focus with a single photograph. To obtain a completely focused image, the extended depth of focus (EDF) imaging technique was adopted, using the Nikon NIS Elements imaging software package (ver. 4 or ver. 5). With this technique, a series of z-stacked individual images were acquired at approximately equal-distance focal steps. For spheroidal objects, for example, between 5 and 15 "confocal" images were acquired, depending on the complexity of morphological features that need to be incorporated.

From the individual confocal images, various combinations of images representing the lower hemisphere, equatorial zone, and upper hemispheres of a spheroidal object can be combined into new, single "focused images", as these combinations help avoid stacking or overlapping cell surface features of the two hemispheres. For the images used in Fig. 3 (D and E; GSC24380e, thin section no. 2) in the main paper, for example, a total of 12 individual confocal images (Suppl-Fig. 1A–L) were obtained for a CUB Type 2 object; image L is a combination of A–C, showing the

morphological features of one “polar area”; image N (see also Fig. 3B in the main paper) is a combination of D–G, representing the “equatorial zone” through the center of the object; image O combines A–G to illustrate one hemisphere, whereas image P (see also Fig. 3C in the main paper) combines G–L to show the other hemisphere. The overall darkness and contrast were adjusted using Adobe Photoshop or Corel PhotoPaint.
